# Balance rehabilitation and Long Covid syndrome: effectiveness of thermal water treatment vs. home-based program

**DOI:** 10.3389/fresc.2025.1588940

**Published:** 2025-06-27

**Authors:** Maria Chiara Maccarone, Paola Contessa, Edoardo Passarotto, Gianluca Regazzo, Stefano Masiero

**Affiliations:** ^1^ Department of Neuroscience, Rehabilitation Unit, University of Padua, Padua, Italy; ^2^ Physical Medicine and Rehabilitation School, Department of Neuroscience, University of Padua, Padua, Italy

**Keywords:** balance impairments, balance rehabilitation, home-based rehabilitation, spa-resort rehabilitation, Long Covid, water-based exercise, COVID-19, spa therapy

## Abstract

**Introduction:**

Balance concerns are increasingly recognized as a common presentation in patients with Long Covid. This study investigates the effects of two distinct rehabilitation programs on balance in a cohort of sixty participants experiencing medium-to-long-term symptoms following SARS-CoV-2 infection.

**Methods:**

Individuals were enrolled and randomly assigned to either a spa resort rehabilitation program or a supervised home-based rehabilitation program. The study assessed balance and proprioception by analyzing the center of pressure trajectory during a standing task performed with eyes open and closed before, after, and at a 3- and 6-month follow-up after the rehabilitation program.

**Results:**

Results indicated that, right after rehabilitation, participants who enrolled in the home-based program demonstrated more significant improvements in mean stay time and in the standard deviation of oscillations in the antero-posterior direction than those who enrolled in a spa-resort program. On the other hand, at the 3-month follow-up, individuals who enrolled in the spa-resort program exhibited improvements in the standard deviation of oscillations in the antero-posterior direction, indicating ongoing benefits over time.

**Discussion:**

These findings suggest that appropriate rehabilitation programs, whether at home or in spa resorts, can contribute to enhancing overall physical function in these patients.

## 1 Introduction

Coronavirus Diseases 2019 (COVID-19), caused by the severe acute respiratory syndrome coronavirus 2 (SARS-CoV-2), manifests with a wide range of symptoms, from mild conditions such as fever and cough to severe manifestations such as pneumonia and acute respiratory distress syndrome (1). Although most patients recover from the acute phase of the illness, a significant number (up to 60%) may develop post-COVID-19 conditions, also known as Long COVID (2–4). This condition is characterized by persistent or emerging symptoms that last more than three months after the initial infection, which cannot be attributed to alternative diagnoses (5–7). In particular, a variety of neurological symptoms, including dizziness, vertigo, and balance disorders, have been reported during the course of acute COVID-19 infection, with varying degrees of severity (8). Among patients with long COVID, symptoms of this type have also been reported (9–12). Vertigo was prominently reported in patients with Long COVID in 2020. Neuropathy and sensory-motor disorders have been reported frequently in recent years, particularly in 2023 (13).

The exact mechanisms behind these symptoms remain unclear. It is hypothesized that SARS-CoV-2 may directly affect neural structures or contribute to dysregulation in multiple organ systems involved in balance, such as the cardiovascular and autonomic nervous systems (14–16). The virus may also cause direct damage to neural structures, potentially leading to conditions like encephalitis, neuron and nerve tissue damage, toxic infectious encephalopathy, or acute cerebrovascular disease (12, 15, 17, 18). However, it is not yet fully understood whether the virus directly disrupts the vestibular system or if such dysfunction may result from an ongoing infectious process within neural structures (11, 19).

Rehabilitation programs for Long COVID patients should be highly individualized, addressing the specific needs, resources, and abilities of each person (20, 21). Among the several symptoms of Long COVID, balance-related issues such as dizziness, vertigo, and postural instability are particularly concerning due to their significant impact on patients’ quality of life and independence. Therefore, these challenges necessitate careful consideration when designing rehabilitation strategies tailored to this population.

Rehabilitation approaches for Long COVID patients have been implemented in various settings, including home settings and specialized facilities such as health resorts. Home-based interventions have shown promise in alleviating symptoms such as dyspnea and improving overall quality of life (22–24). They also have the added advantage of maintaining continuity of care during periods of restricted mobility or lockdowns. Spa resorts, on the other hand, have been proposed as part of a new healthcare model, serving as innovative rehabilitation environments that offer a combination of specialized care and traditional therapies (25–27). Exercise in thermal, mineral-rich water has potential benefits in managing Long COVID symptoms. Aquatic exercise, in particular, offers specific advantages for addressing balance impairments. The buoyancy of water minimizes joint strain and enables patients to perform movements more easily. Hydrostatic pressure supports circulation and enhances proprioceptive input. Additionally, water resistance facilitates controlled muscle strengthening, thereby improving balance and reducing the risk of falls (28–30). This model not only reduces the burden on hospital systems by redistributing resources, but also promotes social interaction and enhances psychological well-being, crucial for individuals recovering from the prolonged isolation associated with the pandemic (31).

To date, however, there is still a lack of a comprehensive approach to ensure an objective evaluation of balance-related systems in patients with Long COVID, and balance issues are often overlooked in patients who present other symptoms related to Long COVID. This study investigates the effect on balance in patients with Long COVID syndrome who presented at a rehabilitation service for issues related to COVID-19 infection, such as fatigue, respiratory, cognitive, or psychological disturbances. Specifically, two different treatments were investigated: a rehabilitation program conducted in a spa resort and a home-based program.

## 2 Materials and methods

### 2.1 Participants

In this randomized trial, 103 patients of both sexes, experiencing medium to long-term symptoms following SARS-CoV-2 infection, were evaluated. The patients visited the Rehabilitation Unit of the Azienda Ospedale Università Padova, Padova, Italy, for symptoms related to Long Covid syndrome not attributable to other conditions. Specifically, they presented with fatigue, respiratory, cognitive, or psychological disturbances. A researcher from the Rehabilitation Unit of the Azienda Ospedale Università Padova performed an initial outpatient evaluation, during which the inclusion and exclusion criteria for the study were assessed. Sixty (60) participants agreed to participate in the study, and were randomly assigned to either a spa resort rehabilitation intervention (Group A) or a home-based rehabilitation program (Group B) on a 1:1 ratio. Recruitment took place from January 2023 to April 2023. Part of the population in this study was the same as that in a previous study, currently under review, which assessed various clinical parameters to evaluate quality of life, pain, and motor, respiratory, and psychological function before and after treatment.

Eligibility criteria included participants aged between 18 and 75 years who had a confirmed COVID-19 infection, with a positive test for SARS-CoV-2 nucleic acid in respiratory samples (e.g., oropharyngeal swabs), between 3 and 18 months prior to the initial evaluation. Other criteria included a Fatigue Assessment Scale (FAS) score of ≥22 or a Modified Medical Research Council (MRC) Dyspnea Scale score of ≥2, Beck Depression Inventory (BDI) scores >14 and/or Beck Anxiety Inventory (BAI) scores ≥8, the presence of cognitive symptoms unrelated to other conditions, and no participation in any thermal mineral-rich therapy cycles in the six months preceding the study. Exclusion criteria included the inability to comprehend or sign the informed consent form, current SARS-CoV-2 nucleic acid positivity in respiratory samples, epilepsy, severe psychiatric disorders, neoplasms, pregnancy, skin infections, open wounds, systemic inflammation, and heart, liver, respiratory, or kidney failure, urinary or fecal incontinence, and diagnosis of neurological diseases that may affect balance.

This study was conducted in accordance with the Declaration of Helsinki and received approval from the Ethics Committee of Azienda Ospedale Università Padova (359n/AO/23). All participants reviewed, understood, and signed the informed consent form.

### 2.2 Intervention

Patients in Group A engaged in a 5-week rehabilitation program with two sessions per week, totaling 10 sessions, conducted at a spa resort. Each session comprised a series of four treatments: 10 min of thermal mineral-rich aerosol therapy; 30 min of land-based motor, respiratory, balance, and proprioceptive exercises in the gym; 30 min of motor rehabilitation and balance training in thermal mineral-rich water; and 30 min of cognitive enhancement therapy. Balance training included specific exercises such as using balance boards and stability cushions to improve postural control and coordination. Water-based and dry-land sessions were conducted in small groups, with a maximum of four patients per group. The thermal mineral-rich waters used were salso-bromoiodic waters from the Thermal Basin of Abano Terme, Italy, with a temperature of around $36^{\circ }$C in the pool. A physiotherapist from the spa resort, trained by the researchers from the Rehabilitation Unit of the Azienda Ospedale Università Padova supervised the motor, respiratory, proprioceptive, and balance exercises both in the pool and in the gym.

Group B participated in a similar 5-week program, consisting of two sessions per week, but conducted at home. This program included physiotherapist-led live exercise sessions and neurocognitive support sessions aimed at enhancing memory, attention, and problem-solving skills (such as puzzles, crosswords, Sudoku, and memory games). Sessions were held twice a week. During the recruitment visit, patients were instructed on motor, respiratory, balance, and proprioceptive exercises. They received multimedia materials, including summary sheets and instructional videos, to facilitate adherence and correct exercise execution. Supervised home-based exercises mirrored those given to Group A, and each exercise session lasted 1 h. The balance exercises were similar to those performed at the spa resort and also used equipment such as balance boards and stability cushions. Patients were also provided with a list of self-administered neurocognitive exercises, to be performed for 30 min twice a week, following minimal training.

### 2.3 Assessment

Patients in both groups were assessed before treatment (T0), right after treatment (T1), and during follow-up evaluations at approximately 3 months (T2) and 6 months (T3) after completing the treatment. Static balance was assessed by asking participants to stand barefoot on a force platform (Argoplus, Fremslife S.r.l., Genova, Italy) for 40 s with their arms relaxed on the side of the body and with their feet parallel and heels together. All participants were tested in two conditions at all measurement points: first with their eyes open (EO) while looking at a target placed at eye level and then with their eyes closed (EC).

The force platform measured the position of the center of pressure (COP) at a frequency of 100 Hz. Static balance parameters were estimated by the force platform proprietary software. These included: the sway path, or SP (mm/s), which is the length of the COP trajectory divided by measurement time; the sway area, or SA (${\mathrm{mm}}^{2}$/s), which is the time integral of the area swept by the COP trajectory with respect to the platform center divided by measurement time; the ratio between SA and SP (mm); the amplitude of the oscillations of the COP trajectory in the antero-posterior (AP) and medio-lateral (ML) directions, or SD OAP and SD OLL (mm); the ellipse area, or AE (${\mathrm{mm}}^{2}$), which is the area of the ellipse containing 95% of the COP samples; the mean spatial distance, or SD (mm), which is the average displacement of the COP trace between one peak of the sway density curve and the next; and the mean stay time, or ST (s), which is the average time spent by the COP trace in a neighboring of each peak of the sway density curve (32–34).

### 2.4 Statistical analysis

A paired Wilcoxon test was used to test significant differences in parameters between the EO and EC conditions for each group using alpha = 0.05. Post-hoc analyses were performed using mixed effects regression models to measure participants’ improvement between time points (i.e., T0 vs. T1, T1 vs. T2) while accounting for possible biases due to participant characteristics and variability in time measures. In the models, parameters were entered as dependent variables and predicted by *time*, *Group*, time*Group interaction, participants’ *Age* and *Body Mass Index (BMI)*. *Day*, which measures the number of days between time points, was additionally included in the model. Random intercepts for each participant were modeled to account for inter-individual differences at baseline. *Age* and *BMI* were standardized across participants while *Age* was normalized to the 0–1 range. Group coded Group A = 1 (spa resort rehabilitation treatment) and Group B = 0 (home-based exercise program). Therefore, time*Group interaction coefficients measured the improvement of Group A relative to Group B. EO and EC conditions were analyzed independently. Data from T3 were not considered in the statistical analyses due to the substantial attrition.

## 3 Results

Sixty individuals of both genders who were experiencing medium-to-long-term effects following SARS-CoV-2 infection were considered in the analysis. Of these, thirty individuals received treatment at a spa resort (Group A, 17 females and 13 males, age 50 ± 14 years), and 30 individuals participated in a home-based exercise program (Group B, 15 females and 15 males, 57 ± 14). Patients’ demographic data are reported in Table 1.

**Table 1:** Participants’ data.

Parameters	Experimental group	Control	Total	*p*-value
Number of participants	30	30	60	
Number of females	17/30	15/30	33/60	0.796
Age (years)	50 (14)	57 (14)	54 (14)	0.045*
Height (cm)	169.5 (8.3)	170.7 (8.7)	170.0 (8.5)	0.678
Weight (kg)	75.9 (15.7)	76.0 (15.8)	75.9 (15.6)	0.636
BMI	26.3 (4.7)	26.0 (4.7)	26.0 (4.7)	0.600

Significant differences between groups were tested with a Wilcoxon test for age, height, weight, and BMI, and with a chi-square test of independence for the proportions of males and females in the two groups.

Five of the subjects recruited (all from Group B) participated in only one session (T0) and were therefore considered dropouts. Technical issues during testing prevented us from using data collected from five subjects from Group A and two subjects from Group B. Furthermore, data from four subjects from Group A was discarded due to poor compliance during testing, Therefore, twenty-one subjects for Group A and twenty-three subjects from Group B were considered for further analysis.

**Figure 1:**
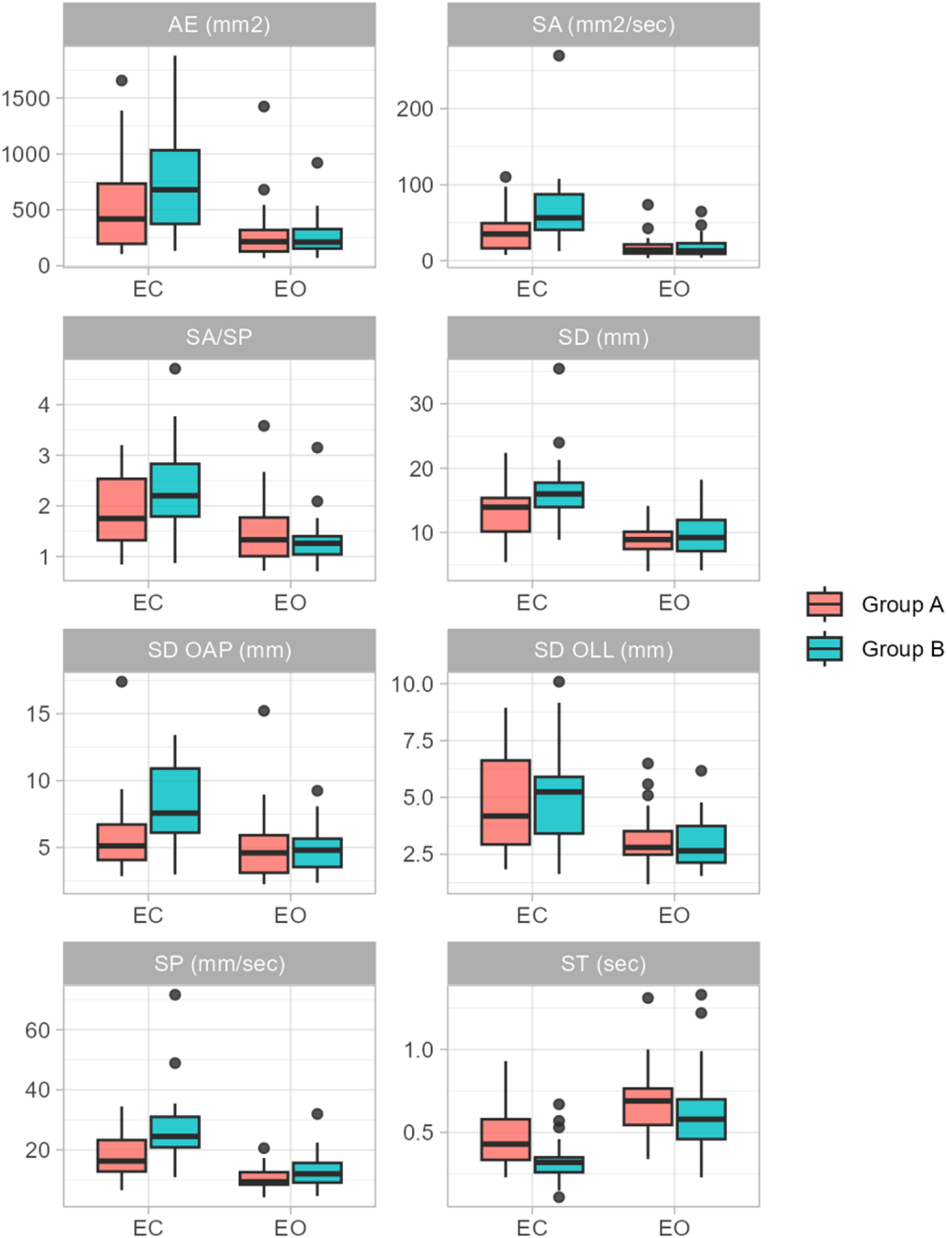
Plot of parameter values for Group A and Group B in the EO and EC condition before treatment (at T0). The plots include values from *n* = 23 individuals for Group B and *n* = 21 individuals for Group A.

Before treatment (T0), values of all parameters increased from the EO to the EC condition for both groups (see Table 2 and Figure 1).

**Table 2:** Values of parameters for Group A and Group B in the EO and EC condition pre-treatment (at T0).

Parameters	Group A (*n* = 21)		Group B (*n* = 22)	
	EO	EC	EO	EC
SP (mm/s)	10.81 ± 4.01	18.13 ± 7.88*	13.23 ± 6.49	26.99 ± 13.41*
SA (${\mathrm{mm}}^{2}$/s)	17.98 ± 15.14	37.89 ± 27.69*	19.41 ± 15.21	70.17 ± 55.68*
SA/SP	1.51 ± 0.70	1.89 ± 0.71*	1.33 ± 0.53	2.38 ± 0.97*
SD OAP (mm)	5.10 ± 2.80	5.93 ± 3.05*	4.97 ± 1.92	8.16 ± 3.11*
SD OLL (mm)	3.15 ± 1.29	4.75 ± 2.21*	3.00 ± 1.17	5.15 ± 2.13*
AE (${\mathrm{mm}}^{2}$)	298.52 ± 289.62	511.00 ± 413.86*	257.88 ± 194.90	757.88 ± 474.86*
ST (s)	0.68 ± 0.22	0.48 ± 0.19*	0.62 ± 0.28	0.33 ± 0.13*
SD (mm)	8.93 ± 2.34	12.92 ± 4.26*	9.81 ± 3.36	16.64 ± 5.59*

The asterisks indicate significant differences (p < 0.05) between the EO and EC conditions.

**Figure 2:**
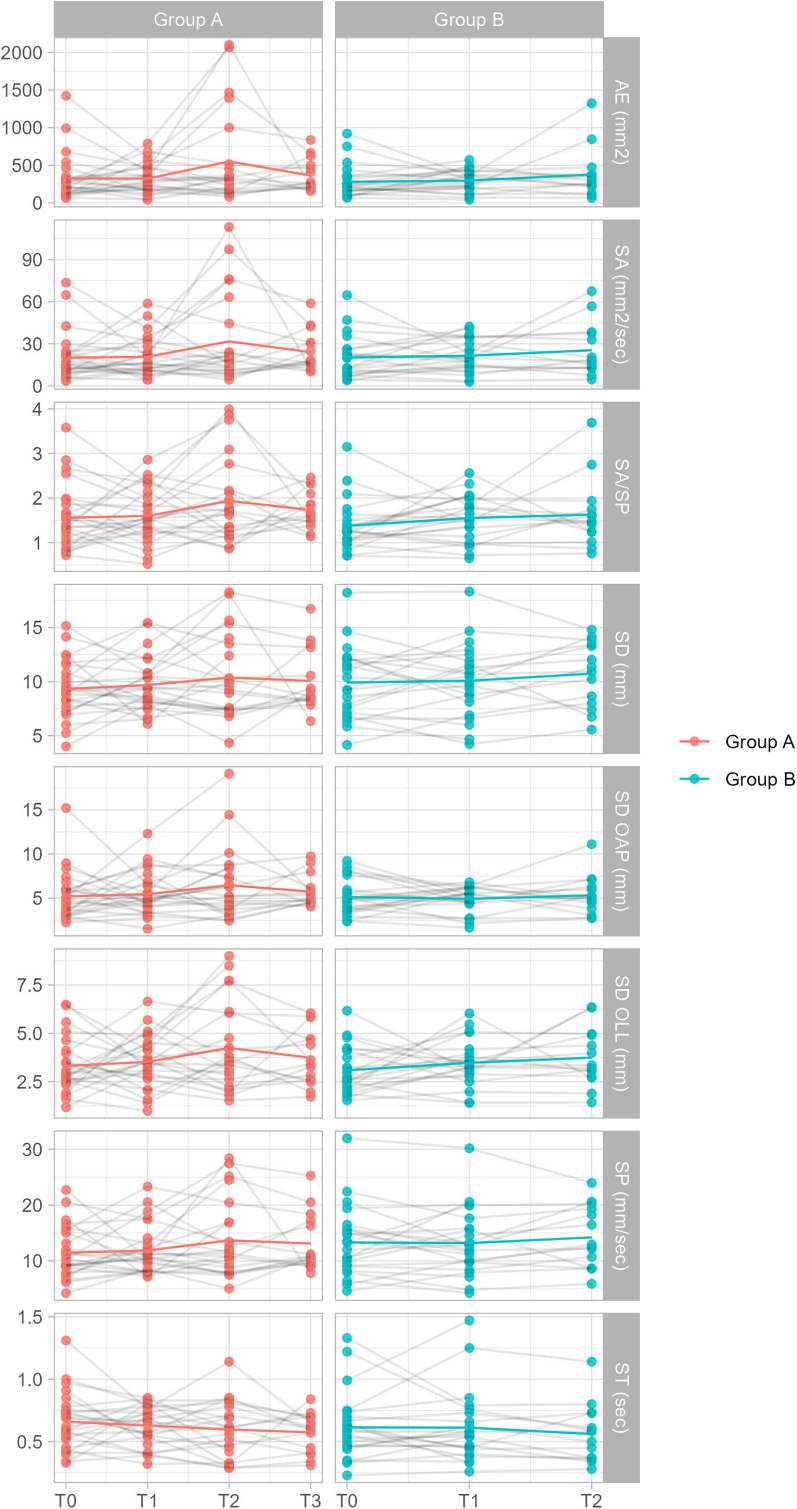
Plot of parameter values for both groups in the EO condition. The plots include values from *n* = 48 individuals. Note: statistical analysis was performed accounting for confounding variables.

**Figure 3:**
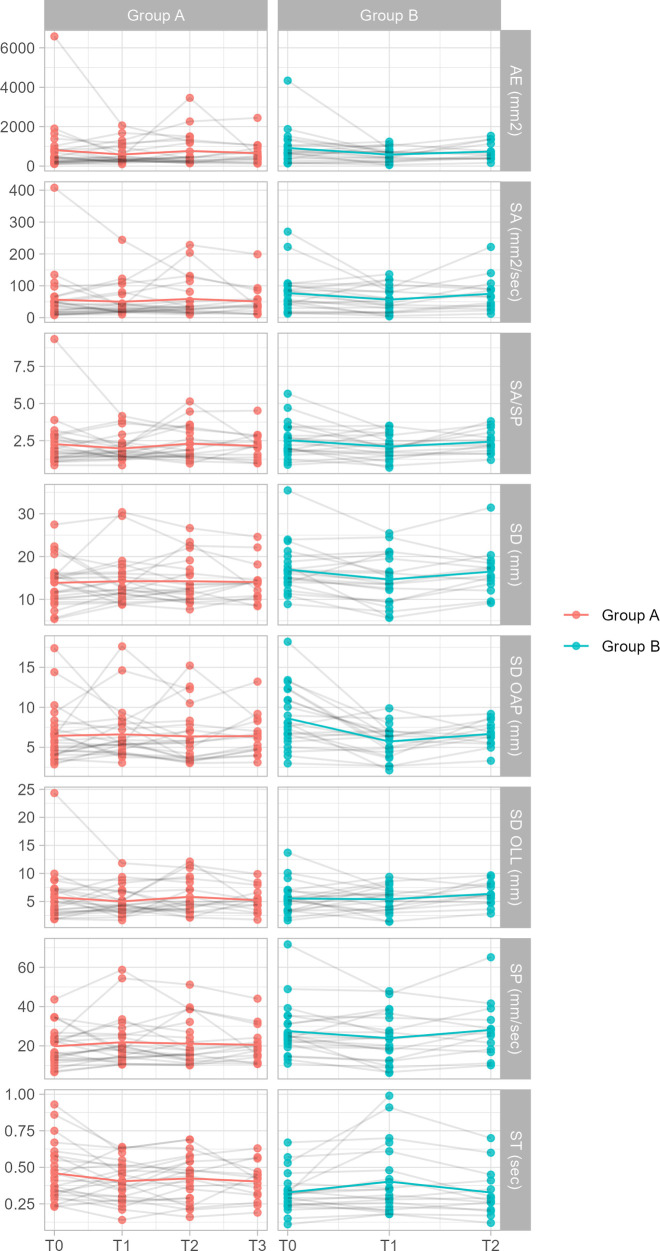
Plot of parameter values for both groups in the EC condition. The plots include values from *n* = 47 individuals. Note: statistical analysis was performed accounting for confounding variables.

Post hoc analyses performed using mixed effects regression models revealed significant effects or interactions only in the EC condition and only for the SD OAP and ST parameters (see Tables 3, 4 and Figures 2, 3). Specifically:

• A significant and negative Time effect in SD OAP values, suggesting a decrease in the amplitude of the antero-posterior oscillations from T0 to T1 for Group B;
• A significant and positive Group effect in ST values, suggesting a group difference at T0 with Group A showing greater values of mean stay time compared to Group B;
• A significant and positive Time effect in ST values, suggesting an increase in the value of mean stay time from T0 to T1 for Group B;
• A significant and negative *time:Group* interaction in ST values measured at T0 and T1, β = −0.119, *p* < 0.05, suggesting that, compared to Group B, values of ST for Group A decreased from T0 and T1.

**Table 3:** Summary of the mixed effect regression models measuring Group differences between T0 and T1 in the EO condition (*N* = 44).

DV	Intercept	Age	BMI	Day	Time	Group	Time:Group
SP (mm/s)	12.516*	2.374*	−0.832	2.829	−0.895	−1.05	0.214
SA (${\mathrm{mm}}^{2}$/s)	17.975*	5.34*	−3.559*	7.873	−0.307	1.314	−2.259
SA/SP	1.307*	0.13	−0.158*	0.379	0.1	0.229	−0.243
SD OAP (mm)	4.966*	0.135	−0.342	1.105	−0.406	0.145	0.251
SD OLL (mm)	2.884*	0.388*	−0.151	0.767	0.229	0.369	−0.367
AE (${\mathrm{mm}}^{2}$)	247.128*	45.913	−44.704	182.905	−21.094	61.211	−58.185
ST (s)	0.643*	−0.09*	0.066*	−0.172	0.051	0.02	−0.017
SD (mm)	9.443*	1.235*	−0.527	1.98	−0.315	−0.182	−0.144

**Table 4:** Summary of the mixed effect regression models measuring Group differences between T0 and T1 in the EC condition (*N* = 44).

DV	Intercept	Age	BMI	Day	Time	Group	Time:Group
SP (mm/s)	25.587*	4.699*	−1.211	4.344	−4.585	−6.045	4.539
SA (${\mathrm{mm}}^{2}$/s)	66.149*	15.303*	−8.309	−1.698	−14.36	−24.045*	18.257
SA/SP	2.329*	0.271*	−0.291*	−0.142	−0.292	−0.376	0.312
SD OAP (mm)	8.08*	0.416	−0.493	1.483	−2.977*	−2.064*	2.868
SD OLL (mm)	4.97*	0.778*	−0.601*	−0.167	0.206	−0.026	−0.239
AE (${\mathrm{mm}}^{2}$)	734.101*	116.758*	−119.467*	115.172	−226.739*	−195.851	177.732
ST (s)	0.351*	−0.071*	0.033	−0.101	0.109*	0.108*	−0.119*
SD (mm)	16.065*	2.026*	−0.719	3.306	−3.111*	−2.553	2.157

No significant Group or *time:Group* effects were observed in the OC condition at any time point.

When analyzing T1 and T2 time points, post hoc analyses revealed only a significant and negative *time:Group* interaction in SD OAP values measured at T1 and T2 in the EC condition, β = −1.826, *p* < 0.05 (see Tables 5, 6). This suggests that the amplitude of SD OAP values decreased significantly from T1 to T2 for Group A compared to Group B, whereas values for Group B did not change significantly from T0 and T1.

**Table 5:** Summary of the mixed effect regression models measuring Group differences between T1 and T2 in the EO condition (*N* = 35).

DV	Intercept	Age	BMI	Day	Time	Group	Time:Group
SP (mm/s)	12.607*	2.41*	−0.736	−3.661	0.702	−0.371	0.267
SA (${\mathrm{mm}}^{2}$/s)	19.753*	4.957	−3.764	−3.353	4.758	0.966	2.25
SA/SP	1.51*	0.086	−0.09	0.162	0.126	0.105	0.088
SD OAP (mm)	4.892*	−0.069	0.294	1.144	0.42	0.858	0.028
SD OLL (mm)	3.327*	0.326	−0.179	−1.172	0.289	0.206	0.237
AE (${\mathrm{mm}}^{2}$)	284.432*	25.098	−33.141	−186.839	86.031	36.732	74.571
ST (s)	0.623*	−0.104*	0.042	0.125	−0.028	−0.017	0.025
SD (mm)	9.802*	1.388*	−0.36	−2.477	0.373	0.044	−0.025

**Table 6:** Summary of the mixed effect regression models measuring Group differences between T1 and T2 in the EC condition (*N* = 34).

DV	Intercept	Age	BMI	Day	Time	Group	Time:Group
SP (mm/s)	23.766*	4.578*	−2.297	−13.045*	2.868	−1.913	−4.283
SA (${\mathrm{mm}}^{2}$/s)	51.82*	14.346*	−10.435	−95.673*	16.947	−6.129	−10.616
SA/SP	1.974*	0.214	−0.205	−1.282*	0.387*	−0.048	−0.178
SD OAP (mm)	5.651*	0.341	−0.01	0.666	0.937	1.287	−1.826*
SD OLL (mm)	5.236*	0.522	−0.73*	−3.705*	1.024	−0.419	−0.567
AE (${\mathrm{mm}}^{2}$)	542.253*	79.392	−104.9	−678.572*	173.85	9.242	−94.607
ST (s)	0.395*	−0.068*	0.046	0.131	−0.053*	0.003	0.088
SD (mm)	14.596*	1.972*	−1.088	−5.278*	1.279	−0.335	−1.771

No significant Group or *time:Group* effects were observed in the OC condition at any time point.

## 4 Discussion

The findings from this study provide insights into the effects of rehabilitation interventions on balance-related parameters in patients with Long Covid. A literature analysis has shown that in Long Covid syndrome, symptoms affecting various systems (respiratory, postural, motor, neurocognitive, psychological) are often interconnected and co-occurring (13). A previous study that investigated the population of Long Covid patients seeking rehabilitation services at the Azienda Ospedale Università Padova showed that many patients experienced multiple symptoms, with the majority reporting 5 to 8 symptoms simultaneously, most of which were associated with fatigue (35). This is one of the few studies that have investigated the presence of balance impairments in patients receiving rehabilitation due to symptoms associated with Long Covid syndrome. Gervasoni et al. (2022) showed that, when compared to normal ranges, post-Covid patients were significantly more distant from normality in the EO condition compared to the EC condition. These authors suggested that in the EO condition, the subject integrates information from three sensory systems—vision, somatosensory, and vestibular information—while visual feedback is absent in the EC condition. Once the visual component is excluded, patients with Long Covid syndrome seemed to improve their performance, presumably because they relied more on feedback from the lower limbs (36).

In our study, both groups showed significant changes at baseline in values of all parameters from the EO to the EC condition. This suggests that, when visual feedback was removed (i.e., in the EC condition), both groups demonstrated an increased difficulty in balance tasks, contrary to the findings reported by Gervasoni et al. (36). The absence of visual input appears to have contributed to a greater challenge in maintaining stability, which is consistent with the understanding that visual feedback plays a crucial role in postural control (37, 38). Moreover, our results showed a decrease in the amplitude of the antero-posterior oscillations from T0 to T1 for Group B (home-based rehabilitation) and an increase in ST values, reflecting an improvement in stability from T0 to T1. The home-based rehabilitation program, with exercises targeting posture, balance, and proprioception, may have determined more rapid improvements in postural stability. Group A (spa-based rehabilitation) exhibited greater values of mean stay time with eyes closed compared to Group B at T0. This might explain why, at T1, this parameter unexpectedly decreased for Group A compared to Group B. It could be hypothesized that the intensive nature of the spa resort program, while beneficial for the symptoms presented by the patients, may have led to fatigue or other factors affecting balance performance immediately after the treatment. However, when analyzing the T1 and T2 time points, the amplitude of oscillations in the antero-posterior direction decreased significantly from T1 to T2 for Group A compared to Group B in the EC condition. This suggests that Group A experienced further improvements in postural control over time. It can therefore be hypothesized that the improvements observed in the home-based program may have been more rapid but less sustained over time, while patients in the spa resort program may have experienced more gradual improvement in balance control. Indeed, the aquatic environment can play a significant role in balance recovery due to its physical properties (29, 30, 39, 40). Buoyancy, which refers to the upward force exerted by a fluid that opposes the weight of a body submerged in it, reduces the load on the joints. This allows patients to perform movements with a lower risk of injury, particularly beneficial for individuals with musculoskeletal disorders or mobility issues. In addition, the resistance of water, which increases in proportion to the speed of movement, promotes strength and stability. Hydrostatic pressure, the force exerted by a fluid on an object submerged in it, improves circulation and stimulates proprioception, the body’s ability to sense its position and movement in space, essential for maintaining balance (28). Furthermore, the altered visual input in water forces individuals to rely more on their body’s own sensory feedback, such as proprioception and vestibular input, to maintain body control and coordination, stimulating motor adaptation (41, 42).

Previous studies have evaluated the efficacy of exercise and targeted rehabilitation strategies in a wide range of orthopedic and neuromuscular conditions (43–46). To the best of our knowledge, our study is the first study to evaluate the effects on balance of a comprehensive rehabilitation program, that includes thermal water treatment, in patients with Long Covid. Even with a somewhat limited number of participants, results were able to highlight the potential of offering interventions to improve balance for Long Covid patients in non-hospital settings. It also highlighted potential longer-term benefits of thermal water treatments compared to home-based exercises. This study however only followed patients up to six months after treatment. Potential longer-term benefits could be better assessed by following patients for up to a year after the end of treatment. While more research on this aspect would be useful, specialized spa-resort programs seem to have the potential to provide more sustained benefits to patients with Long Covid. Unfortunately, access to such programs is limited to individuals who live in the vicinity of spa-resorts and regions with thermal-water basins. In contrast, home-based programs are easier to implement on a broader scale, may benefit individuals regardless of their place of abode, and are more financially sustainable (47). Indeed, the number of community-based and home-based interventions experienced a rapid increase during resource constrained times like the COVID-19 epidemic in many countries, including those with more fragmented health care systems (47). Ideally, a hybrid model capable of combining scalable, readily deployable home-based protocols with more specialized, targeted spa-resort referrals (for complex patients) could ensure more equitable access across socioeconomic strata.

Overall, a rehabilitation program focused on enhancing balance and proprioception may prove beneficial for this population. Indeed, Long Covid involves a wide range of symptoms affecting various systems, and balance dysfunction, even if subclinical, could contribute to impairments in daily functioning and quality of life. Targeted interventions that address even subtle balance impairments can contribute to improved postural stability, enhanced overall physical functioning, and could lead to a reduction in fall risk. Furthermore, providing rehabilitation in less traditional settings, such as home-based programs or spa resorts, may offer flexibility and accessibility, potentially increasing patient adherence and engagement.

## 5 Conclusions

This study provides insights into the effects of different rehabilitation approaches on balance and postural control in patients with Long Covid. Both the spa resort and home-based rehabilitation programs resulted in improvements in balance-related parameters, with each approach yielding distinct outcomes. The home-based program showed more rapid improvements, while the spa resort program led to more gradual and sustained improvements in postural stability. Importantly, this study highlights the need for further investigation into balance impairments in Long Covid patients to ensure that rehabilitation programs can be tailored to meet the diverse needs of Long Covid patients.

## Data Availability

The raw data supporting the conclusions of this article will be made available by the authors, without undue reservation.

## References

[1] Hu B Guo H Zhou P Shi Z-L. Characteristics of SARS-CoV-2 and COVID-19. Nat Rev Microbiol. (2021) 19:141–54. 10.1038/s41579-020-00459-7. 33024307 PMC7537588

[2] Chen C Haupert SR Zimmermann L Shi X Mukherjee B Fritsche LG. Global prevalence of post-coronavirus disease 2019 (COVID-19) condition or long covid: a meta-analysis and systematic review. J Infect Dis. (2022) 226:1593–607. 10.1093/infdis/jiac136. 35429399 PMC9047189

[3] de Las-Peñas CF Palacios-Ceña D Gómez-Mayordomo V Florencio LL Cuadrado ML Plaza-Manzano G, et al. Prevalence of post-covid-19 symptoms in hospitalized and non-hospitalized covid-19 survivors: a systematic review and meta-analysis. Eur J Intern Med. (2021) 92:55–70. 10.1016/j.ejim.2021.06.009. 34167876 PMC8206636

[4] Lopez-Leon S Wegman-Ostrosky T Perelman C Sepulveda R Rebolledo PA Cuapio A, et al. More than 50 long-term effects of covid-19: a systematic review and meta-analysis. Sci Rep. (2021) 11:16144. 10.1038/s41598-021-95565-8. 34373540 PMC8352980

[5] Davis HE McCorkell L Vogel JM Topol EJ. Long covid: major findings, mechanisms and recommendations. Nat Rev Microbiol. (2023) 21:133–46. 10.1038/s41579-022-00846-2. 36639608 PMC9839201

[6] Michelen M Manoharan L Elkheir N Cheng V Dagens A Hastie C, et al. Characterising long covid: a living systematic review. BMJ Glob Health. (2021) 6:e005427. 10.1136/bmjgh-2021-005427. 34580069 PMC8478580

[7] Venkatesan P. Nice guideline on long covid. Lancet Respir Med. (2021) 9:129. 10.1016/S2213-2600(21)00031-X. 33453162 PMC7832375

[8] Sia J. Dizziness can be an early sole clinical manifestation for covid-19 infection: a case report. J Am Coll Emerg Physicians Open. (2020) 1:1354–6. 10.1002/emp2.12185. 32838388 PMC7404329

[9] Crook H Raza S Nowell J Young M Edison P. Long covid—mechanisms, risk factors, and management. BMJ. (2021) 26:n1648. 10.1136/bmj.n1648. 34312178

[10] Dziȩcioł-Anikiej Z Dakowicz A Dziȩcioł J Kopko S Moskal-Jasińska D Gawlikowska-Sroka A, et al. Balance disorders in people with history of covid-19 in light of posturographic tests. J Clin Med. (2023) 12:4461. 10.3390/jcm12134461. 37445496 PMC10342615

[11] Ludwig S Schell A Berkemann M Jungbauer F Zaubitzer L Huber L, et al. Post-covid-19 impairment of the senses of smell, taste, hearing, and balance. Viruses. (2022) 14:849. 10.3390/v14050849. 35632590 PMC9145380

[12] Stefanou M-I Palaiodimou L Bakola E Smyrnis N Papadopoulou M Paraskevas GP, et al. Neurological manifestations of long-covid syndrome: a narrative review. Ther Adv Chronic Dis. (2022) 17:20406223221076890. 10.1177/20406223221076890. PMC8859684 35198136

[13] Maccarone MC Coraci D Regazzo G Sarandria N Scanu A Masiero S. Evolution of musculoskeletal symptoms in long covid syndrome: a lexical analysis to approach requirements for an interdisciplinary management. Joint Bone Spine. (2024) 91:105623. 10.1016/j.jbspin.2023.105623. 37487957

[14] Baker AME Maffitt NJ Vecchio AD McKeating KM Baker MR Baker SN, et al. Neural dysregulation in post-covid fatigue. Brain Commun. (2023) 5:fcad122. 10.1093/braincomms/fcad122. 37304792 PMC10257363

[15] Blitshteyn S Whitelaw S. Postural orthostatic tachycardia syndrome (pots) and other autonomic disorders after covid-19 infection: a case series of 20 patients. Immunol Res. (2021) 69:205–11. 10.1007/s12026-021-09185-5. 33786700 PMC8009458

[16] Dani M Dirksen A Taraborrelli P Torocastro M Panagopoulos D Sutton R, et al. Autonomic dysfunction in “long covid”: rationale, physiology and management strategies. Clin Med (Lond). (2021) 21:e63–7. 10.7861/clinmed.2020-0896. 33243837 PMC7850225

[17] Lavienraj Premraj NVK Briggs J Seal SM Battaglini D Fanning J Suen J, et al. Mid and long-term neurological and neuropsychiatric manifestations of post-covid-19 syndrome: a meta-analysis. J Neurol Sci. (2022) 15:120162. 10.1016/j.jns.2022.120162. PMC8798975 35121209

[18] Wu Y Xu X Chen Z Duan J Hashimoto K Yang L, et al. Nervous system involvement after infection with covid-19 and other coronaviruses. Brain Behav Immun. (2020) 87:18–22. 10.1016/j.bbi.2020.03.031. 32240762 PMC7146689

[19] Pazdro-Zastawny K Dorobisz K Misiak P Kruk-Krzemień A Zatoński T. Vestibular disorders in patients after covid-19 infection. Front Neurol. (2022) 20:1956515. 10.3389/fneur.2022.956515. PMC9531925 36203969

[20] DeMars J Brown DA Angelidis I Jones F McGuire F O’Brien KK, et al. What is safe long covid rehabilitation? J Occup Rehabil. (2022) 33:227–30. 10.1007/s10926-022-10075-2. PMC9628454 36315323

[21] Swarnakar R Yadav SL. Rehabilitation in long covid-19: a mini-review. World J Methodol. (2022) 12:235–45. 10.5662/wjm.v12.i4.235. 36159093 PMC9350732

[22] Elyazed TIA Alsharawy LA Salem SE Helmy NA El-Hakim AAE-MA. Effect of home-based pulmonary rehabilitation on exercise capacity in post covid-19 patients: a randomized controlled trail. J Neuroeng Rehabil. (2024) 21:40. 10.1186/s12984-024-01340-x. 38528512 PMC10964649

[23] McGregor G Sandhu H Bruce J Sheehan B McWilliams D Yeung J, et al. Clinical effectiveness of an online supervised group physical and mental health rehabilitation programme for adults with post-covid-19 condition (regain study): multicentre randomised controlled trial. BMJ. (2024) 384:e076506. 10.1136/bmj-2023-076506. 38325873 PMC11134408

[24] Reeves JM Spencer LM Tsai L-L Baillie AJ Han Y Leung RWM, et al. Effect of a 4-week telerehabilitation program for people with post-covid syndrome on physical function and symptoms: protocol for a randomized controlled trial. Phys Ther. (2024) 104:pzae08. 10.1093/ptj/pzae080. PMC11443032 38943360

[25] Maccarone MC Magro G Tognolo L Masiero S. Post covid-19 persistent fatigue: a proposal for rehabilitative interventions in the spa setting. Int J Biometeorol. (2021) 65:2241–3. 10.1007/s00484-021-02158-1. 34086142 PMC8175920

[26] Maccarone MC Masiero S. Spa therapy interventions for post respiratory rehabilitation in covid-19 subjects: does the review of recent evidence suggest a role? Environ Sci Pollut Res Int. (2021) 28:46063–6. 10.1007/s11356-021-15443-8. 34273080 PMC8286038

[27] Masiero S Maccarone MC. Health resort therapy interventions in the covid-19 pandemic era: what next? Int J Biometeorol. (2021) 65:1995–7. 10.1007/s00484-021-02134-9. 33880643 PMC8057917

[28] Becker BE. Aquatic therapy: scientific foundations and clinical rehabilitation applications. PM R. (2009) 1:859–72. 10.1016/j.pmrj.2009.05.017. 19769921

[29] Grishechkina IA Lobanov AA Andronov SV Rachin AP Fesyun AD Ivanova EP, et al. Long-term outcomes of different rehabilitation programs in patients with long covid syndrome: a cohort prospective study. Eur J Transl Myol. (2023) 33:11063. 10.4081/ejtm.2023.11063. 37052043 PMC10388602

[30] Lobanov AA Grishechkina IA Andronov SV Barashkov GN Popov AI Fesyun AD, et al. Can aquatic exercises contribute to the improvement of the gait stereotype function in patients with long covid outcomes? Eur J Transl Myol. (2022) 32:10698. 10.4081/ejtm.2022.10698. 35833897 PMC9580543

[31] Maccarone MC Magro G Solimene U Scanu A Masiero S. From in vitro research to real life studies: an extensive narrative review of the effects of balneotherapy on human immune response Sport Sci Health. (2021) 17:817–35. 10.1007/s11332-021-00778-z. 34035862 PMC8136372

[32] Baratto L Morasso PG Re C Spada G. A new look at posturographic analysis in the clinical context: sway-density versus other parameterization techniques. Motor Control. (2002) 6:246–70. 10.1123/mcj.6.3.246. 12122219

[33] Gallamini M Piastra G Porzio D Ronchi M Scoppa F Bertora F. Instrumental assessment of balance functional performance. A numerical score to discriminate defective subjects: a retrospective study. J Nov Physiother. (2016) 6:1000305. 10.4172/2165-7025.1000305.

[34] Scoppa F Capra R Gallamini M Shiffer R. Clinical stabilometry standardization: basic definitions–acquisition interval–sampling frequency. Gait Posture. (2013) 37:290–2. 10.1016/j.gaitpost.2012.07.009. 22889928

[35] Maccarone MC Coraci D Regazzo G Masiero S. Symptoms patterns and health-related quality of life in a real-life cohort of long-covid patients: complexity to optimize rehabilitation treatment. Am J Phys Med Rehabil. (2025) 104:231–5. 10.1097/PHM.0000000000002578. 38958179

[36] Gervasoni F LoMauro A Ricci V Salce G Andreoli A Visconti A, et al. Balance and visual reliance in post-covid syndrome patients assessed with a robotic system: a multi-sensory integration deficit. Neurol Sci. (2022) 43:85–8. 10.1007/s10072-021-05647-8. 34613505 PMC8493357

[37] Luo H Wang X Fan M Deng L Jian C Wei M, et al. The effect of visual stimuli on stability and complexity of postural control. Front Neurol. (2018) 9:48. 10.3389/fneur.2018.00048. 29472888 PMC5809403

[38] Paramento M Passarotto E Maccarone MC Agostini M Contessa P Rubega M, et al. Neurophysiological, balance and motion evidence in adolescent idiopathic scoliosis: a systematic review. PLoS One. (2024) 19:e0303086. 10.1371/journal.pone.0303086. 38776317 PMC11111046

[39] Marco RD Pistonesi F Cianci V Biundo R Weis L Tognolo L, et al. Effect of intensive rehabilitation program in thermal water on a group of people with parkinson’s disease: a retrospective longitudinal study. Healthcare (Basel). (2022) 10:368. 10.3390/healthcare10020368. 35206982 PMC8871929

[40] Volpe D Giantin MG Manuela P Filippetto C Pelosin E Abbruzzese G, et al. Water-based vs. non-water-based physiotherapy for rehabilitation of postural deformities in parkinson’s disease: a randomized controlled pilot study. Clin Rehabil. (2017) 31:1107–15. 10.1177/0269215516664122. 27512099

[41] Saleh MSM Rehab NI Aly SMA. Effect of aquatic versus land motor dual task training on balance and gait of patients with chronic stroke: a randomized controlled trial. NeuroRehabilitation. (2019) 44:485–92. 10.3233/NRE-182636. 31256082

[42] Zughbor N Alwahshi A Abdelrahman R Elnekiti Z Elkareish H Gabor MG, et al. The effect of water-based therapy compared to land-based therapy on balance and gait parameters of patients with stroke: a systematic review. Eur Neurol. (2021) 84:409–17. 10.1159/000517377. 34274928

[43] Farì G Paolo SD Ungaro D Luperto G Farì E Latino F. The impact of covid-19 on sport and daily activities in an Italian cohort of football school children. Int J Athl Therapy Train. (2021) 26:274–8. 10.3390/clinpract14060216.

[44] Iaconisi GN Mancini R Ricci V Donati D Sconza C Marvulli R, et al. Biochemical mechanisms and rehabilitation strategies in osteoporosis-related pain: a systematic review. Clin Pract. (2024) 14:2737–58. 10.3390/clinpract14060216. 39727804 PMC11674043

[45] Manocchio N Ljoka C Buttarelli L Giordan L Sorbino A Foti C. Early motor and respiratory re-education in patients hospitalized for covid-19. Adv Rehab. (2025) 39:29–45. 10.5114/areh.2025.149344.

[46] Musumeci A Pranovi G Masiero S. Patient education and rehabilitation after hip arthroplasty in an italian spa center: a pilot study on its feasibility. Int J Biometeorol. (2018) 62:1489–96. 10.1007/s00484-018-1548-9. 29748911

[47] Carta MG Orru’ G Littera R Firinu D Chessa L Cossu G, et al. Comparing the responses of countries and national health systems to the covid-19 pandemic- a critical analysis with a case-report series. Lit Rev. (2023) 27:7868–80. 10.26355/eurrev_202308_33442. 37667964

